# Acceleration of inferred neural responses to oddball targets in an individual with bilateral amygdala lesion compared to healthy controls

**DOI:** 10.1038/s41598-023-41357-1

**Published:** 2023-09-04

**Authors:** Aslan Abivardi, Christoph W. Korn, Ivan Rojkov, Samuel Gerster, Rene Hurlemann, Dominik R. Bach

**Affiliations:** 1 Computational Psychiatry Research, Department of Psychiatry, Psychotherapy and Psychosomatics, Psychiatric University Hospital Zurich, University of Zurich, 8032 Zurich, Switzerland; 2 Wellcome Centre for Integrative Neuroimaging, FMRIB, Nuffield Department of Clinical Neurosciences, University of Oxford, Oxford, OX3 9DU UK; 3 Section Social Neuroscience, Department of General Adult Psychiatry, Heidelberg University, 69115 Heidelberg, Germany; 4 Institute for Systems Neuroscience, University Medical Center Hamburg-Eppendorf, 20246 Hamburg, Germany; 5 Institute for Quantum Electronics, ETH Zurich, 8093 Zurich, Switzerland; 6 Department of Psychiatry, School of Medicine & Health Sciences, Carl von Ossietzky University of Oldenburg, 26160 Bad Zwischenahn, Germany; 7 Hertz Chair for Artificial Intelligence and Neuroscience, University of Bonn, 53012 Bonn, Germany

**Keywords:** Human behaviour, Attention, Amygdala, Neural circuits, Neurophysiology, Neurodevelopmental disorders

## Abstract

Detecting unusual auditory stimuli is crucial for discovering potential threat. Locus coeruleus (LC), which coordinates attention, and amygdala, which is implicated in resource prioritization, both respond to deviant sounds. Evidence concerning their interaction, however, is sparse. Seeking to elucidate if human amygdala affects estimated LC activity during this process, we recorded pupillary responses during an auditory oddball and an illuminance change task, in a female with bilateral amygdala lesions (BG) and in n = 23 matched controls. Neural input in response to oddballs was estimated via pupil dilation, a reported proxy of LC activity, harnessing a linear-time invariant system and individual pupillary dilation response function (IRF) inferred from illuminance responses. While oddball recognition remained intact, estimated LC input for BG was compacted to an impulse rather than the prolonged waveform seen in healthy controls. This impulse had the earliest response mean and highest kurtosis in the sample. As a secondary finding, BG showed enhanced early pupillary constriction to darkness. These findings suggest that LC-amygdala communication is required to sustain LC activity in response to anomalous sounds. Our results provide further evidence for amygdala involvement in processing deviant sound targets, although it is not required for their behavioral recognition.

## Introduction

Unexpected and deviant sounds in natural environments can be a harbinger of imminent threat and request a specialized system for rapid detection, critical to survival across species^1,2^. The mammal brain implements such a system in a hierarchical manner along the auditory pathway, i.e., from brainstem, subcortex and primary auditory cortex to higher-order cortical brain regions^3,4^. Through wide-spread noradrenergic projections, locus coeruleus (LC) can modulate response patterns of cortical sensory regions to salient stimuli^5,6^, while amygdala is thought to be involved in their detection^7^.

LC is a bilateral, primarily noradrenergic (NA) nucleus located in the pons, dorsolaterally to the fourth ventricle, and forms a main hub for the coordination of attentive and task-related cognitive processes^8,9^. Pupil dilation recordings provide an easily accessible route for indirect tracking of the nucleus’ activity across species^10,11^. Chemogenic activation of the LC-NA system, for example, has been demonstrated to increase brain-wide communication in rodents, in particular, in salience and amygdala networks, accompanied by pupil dilation and anxiety-like behavior^12^. The interaction of LC-NA with auditory processing presents a complex and multifaceted picture, as NA has been related to both suppression of neural activity in auditory cortices and enhanced perception of sounds^13,14^ including synchronized neuroplastic effects in both LC and primary auditory cortex^15^.

Parallel to the LC-NA system’s role in coordinating attention, the amygdala, a highly interconnected subcortical structure, can modulate perception and attention by integrating information from salient or relevant stimuli^1,16–18^. Accordingly, clinical lesion studies in humans have suggested weakened processing of negatively arousing stimuli such as aversive words (Anderson and Phelps^19^; although see Bach et al.^20^) and angry faces^21^. Furthermore, amygdala has been implicated in processing non-emotional novel and surprising sensory inputs^7,22^.

Projections from LC to amygdala and vice-versa have been shown to alter each regions’ respective activity and their stimulation evokes anxiety-like behavior in rodents^23,24^. LC-NA system and amygdala dysfunction are speculated to underly human stress-related and affective psychiatric disorders^25–28^. Stronger functional coupling between LC and amygdala in an emotional conflict experiment has been associated with decreased real-life stress tolerability in humans^29^. A recent LC and amygdala pharmacological inactivation study in mice, moreover, has implicated LC-amygdala circuit interaction in control of early olfactory sensory gating^30^. How human LC and amygdala interact during basic behavioral processes such as the detection of potentially threatening auditory events remains unknown.

To investigate if and how amygdala affects LC output during oddball target detection, we recorded pupil response data from a female subject with extensive lesions of bilateral amygdala, caused by calcifications due to a very rare genetic disorder (Urbach-Wiethe syndrome), and from 23 female age-matched healthy controls, while they performed an auditory oddball task. This paradigm, often used to evoke mismatch negativity and P300 potentials in EEG^31^, contrasts rare acoustic events (i.e., oddballs) with common (repeated) sounds, thus providing a means to investigate mechanisms of anomalous sound detection. Both LC^32^ and amygdala^33^ are responsive to oddball over standard sounds in nonhuman primates. We estimated LC output with a previously established formal and quantitative forward model for pupil dilation, which describes pupil responses as the output of a linear time-invariant system that convolves a neural input with a pupillary response function^34,35^. We estimated each participant's individual pupillary response function from responses to a defined illuminance input. We used this function, together with the time-series of pupil responses to oddball sounds, to model LC output in response to these oddballs.

## Materials and methods

### Participants

The main participant for this study was a German-speaking 43-year-old female with bihemispheric and selective amygdala lesions as part of congenital Urbach-Wiethe syndrome traceable to a de novo homozygous missense mutation in exon 7 of extracellular matrix protein 1 gene^36^.

The extensive tissue damage caused by symmetric calcifications throughout almost the entirety of the participant’s amygdalae has been previously mapped using computer tomography^36,37^ and evaluated by an expert neuroanatomist (Karl Zilles). Specifically, “complete destruction of the basolateral amygdala and minor sparing of anterior amygdaloid and ventral cortical amygdaloid parts at a rostral level, as well as lateral and medial parts of the central amygdaloid nucleus and the amygdalohippocampal area at more caudal levels” was reported. Importantly, there was no damage to hippocampus.

Here and in all cited previous studies the participant is referred to as BG with exception of Becker et al.^36^, where she has been referred to as patient 2.

Aged 12, BG suffered an epileptic grand-mal seizure, but had no further history of epilepsy since. BG has average intelligence and mostly intact performance in a neuropsychological test battery^38^. This included normal immediate and delayed memory function, learning efficiency, attention skills, cognitive flexibility and verbal as well as performance IQ. Psychopathological symptoms such as anxiety or depression were absent across assessments. In contrast, BG demonstrates a number of circumscribed social and affective deficits. Specifically, she lacked antero- and retrograde modulatory effects of emotional perceptions on memory encoding^39^, exhibited increased risk-taking as well as limited impairments in phonemic fluency and a visual test of short-term concentration^38^. Moreover, she had a reduced social network size, absent increase of the acoustic startle reflex in response to negative vs. neutral visual emotional stimuli, severe impairment of recognition of fearful faces^36^ and similarly, a lack of prioritization of angry over happy faces^21^. A recent study from the same testing session suggested subtle changes in prosocial motivation^40^. In contrast, she showed normal (facilitated) recall of aversive words in an attentional blink task^20^, normal discrimination between fearful and neutral prosody^41^, and normal recognition of facial expression of surprise, disgust or happiness^39^.

For the current study, BG was tested at the University Hospital of Bonn in April 2017. Her monozygotic twin sister (AM) could not partake in this study due to a treatment-resistant episode of major depression^42^.

For the control group, we recruited 23 age-matched female volunteers (mean age ± SD, 41.87 ± 3.90) from the general population with no reported history of neurological or psychiatric disease. Controls were tested at the University of Zurich from November 2017 until June 2018. All participants (including BG) had normal or corrected to normal eyesight.

BG and 22 of 23 control participants performed a continuous-illuminance task to derive their individual pupillary response function, and to rule out potential pupillary response impairments in BG. All participants then performed an auditory oddball task.

Participants provided written informed consent prior to the experiments. The study was conducted in accord with the Declaration of Helsinki and was approved by local ethics committees in Bonn (Ethikkommission der Medizinischen Fakultät Bonn) and Zurich (Kantonale Ethikkommission Zürich).

### Experimental setup: continuous illuminance task

**Figure 1:**
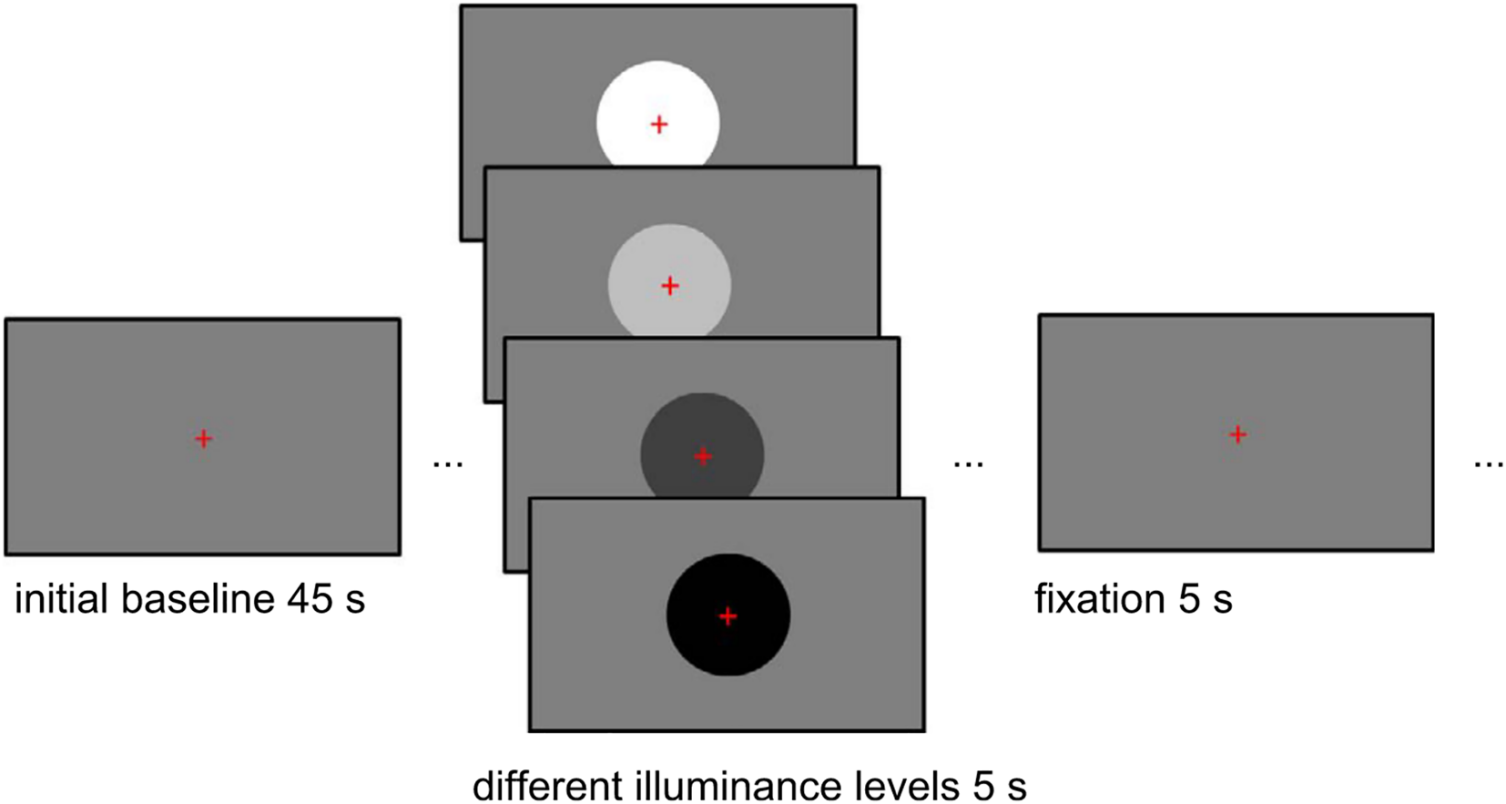
To estimate individual pupillary response functions, a continuous illuminance task^34^ was used. Participants were exposed to a gray baseline screen for 45 s, and then circles with different illuminance levels appeared for 5 s per trial. Participants were instructed to fixate a red cross throughout the task.

We used a previously established continuous illuminance task^34^. The task consisted of five sessions with 24 trials in each session (6 presentations of 4 illuminance levels). Each session began with a 45 s resting period, in which a medium gray screen was shown (46.10 cd/m^2^, 7.30 lx, rgb(128, 128, 128)). In every trial, a circle appeared in the center of the screen and on the medium gray background for 5 s, then disappeared and was followed by 5 s of the background again (Fig. 1). Circles were either black (33.50 cd/m^2^, 5.30 lx, rgb(0, 0, 0)), dark gray (36.70 cd/m^2^, 5.80 lx, rgb(64, 64, 64)), light gray (60.70 cd/m^2^, 9.60 lx, rgb(191, 191, 191)) or white (84.10 cd/m^2^, 13.30 lx, rgb(255, 255, 255)). This leads to illuminance changes for both circle appearance and disappearance resulting in 24 changes from darker to brighter (e.g., appearance of white circle or disappearance of black circle) and 24 changes from brighter to darker (e.g., appearance of dark gray circle or disappearance of light gray circle). Participants were instructed to fixate a red cross during the entire task. No further actions were required during the task. Fixation cross brightness was accounted for when calculating the illuminance levels.

### Experimental setup: auditory oddball task

Sine tones (440 and 660 Hz), served as standard and oddball tones, respectively (length: 50 ms; ramp: 10 ms; loudness: ~ 60 dB balanced across participants). Sounds were delivered via open, circumaural headphones (HD 518, Sennheiser, Wendemark-Wennebostel, Germany). Participants were instructed to fixate a red cross (height/width: 1.478) on a medium gray background (46.1 cd/m^2^, rgb(128, 128, 128), 7.3 lx) for the duration of the task, and to press a key whenever they heard the oddball tone. If they failed to answer before the subsequent tone, the words “No answer” appeared on the screen. The total number of presented oddballs per subject was 30 vs. 120 standard tones. The inter stimulus interval (ISI) between any two tones was set at 2 s.

Task performance (% detected) was close to perfect as expected (mean ± SD, healthy controls (HC): 98.70% ± 3.19%; HC combined with BG: 98.54% ± 3.21%; BG: 95.00%). Reaction times were similar for controls and BG (mean ± SD, HC: 432.53 ms ± 107.18 ms; HC combined with BG: 430.88 ms ± 105.13 ms; BG: 392.82 ms).

### Equipment and recording parameters

Experiments were conducted in a dark, soundproof room. Monitor screen and camera provided background illumination of 3.4 lx. A chin rest was used for the participants and positioned 0.70 m in front of the display (Dell P2012H, viewable image size 20″, aspect ratio set to 5:4, refresh rate: 60 Hz). An EyeLink 1000 system (SR Research, Ottawa, Canada) was used to record pupil diameter and gaze direction. Data was sampled at a rate of 500 Hz; calibration of gaze direction was performed using a nine-point calibration algorithm as implemented in the EyeLink software. Levels of illuminance were measured independently with a luxmeter (Digital Luxmeter MS-1300, Voltcraft, Hirschau, Germany) fixed to the chin rest and positioned at the location of the participant’s eyes during the tasks. Importantly, the exact same equipment was used for all participants.

### Data preprocessing

Visual fixation and saccadic eye movements were identified with the EyeLink 1000 system’s online parsing algorithm, which detects apparent changes in pupil position due to blinking or partial pupil occlusion. All further analyses were performed in MATLAB (Version R2018b, MathWorks, Natick, MA) and using a customized pipeline from the PsychoPhysiological Modelling (PsPM) toolbox (Version 5.0.0, https://bachlab.github.io/PsPM/). Time series were analyzed from the first event until 5 s after the final event. Data from left and right pupils were filtered and combined using the preprocessing pipeline from Kret and Sjak-Shie^43^ as implemented in PsPM. Briefly, using the mean signal from both pupils, this algorithm first determines valid samples by rejecting pupil size values outside a predefined range, filtering out implausibly fast changes in pupil size, removing samples at the edges of temporal data gaps, computing a trendline in order to reject samples too far removed from it, and by excluding small, isolated sample islands. In a second step, the refined signal is smoothed by low-pass filtering (4 Hz), upsampled (from 500 to 1000 Hz) and linearly interpolated (maximum gap between valid samples: 250 ms) to deal with missing data points caused by blinking or head movement while concurrently enhancing temporal resolution and reducing noise.

In both tasks participants were instructed to fixate the screen center, as gaze deviation can distort video-based measurements of pupil size, which depend on gaze angle^44^. We excluded data points when fixation was outside of a circle around the central fixation point with a predetermined visual angle of 4.74°, an approach modified from Korn and Bach^34^, where a square had been used for exclusion. Resulting data were then interpolated and peak normalized for each session.

Baseline pupil diameter was calculated as the mean raw diameter during 500 ms prior to stimulus onset in the auditory task (excluding trials following the oddball tone).

### Modeling individual pupillary dilation response function to illuminance changes

**Figure 2:**
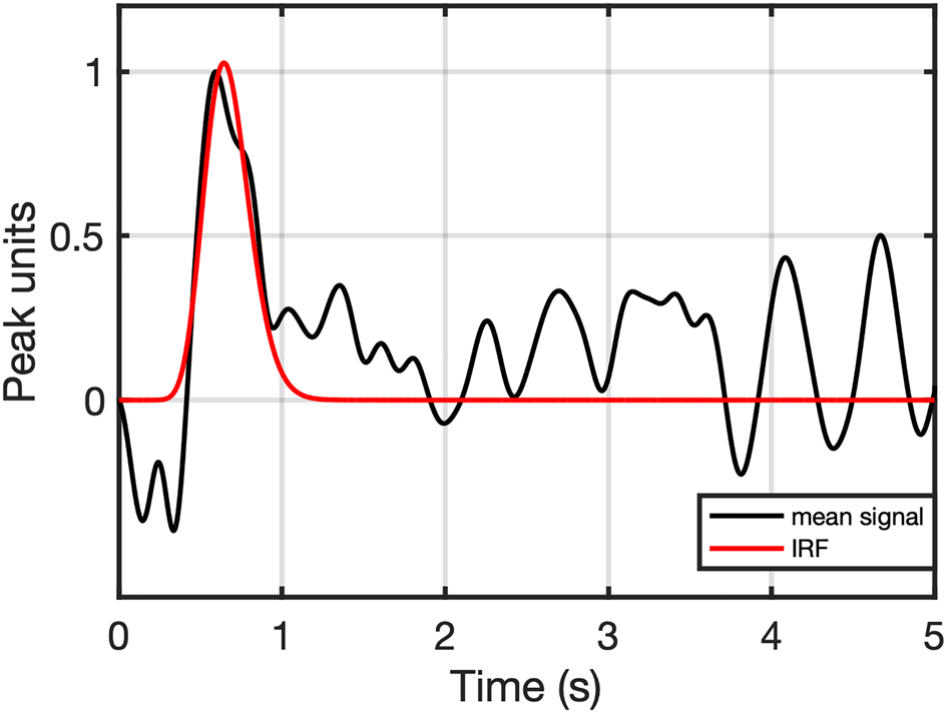
Estimation of individual pupil dilation response function (IRF, exemplified here for BG). The initial peak of the time derivative of the low-pass filtered mean pupil response to rapid darkening of continuous illuminance (black), which induces dilation, was approximated with a gamma probability distribution function (red). Data was zeroed after the initial pupil response of interest (trough after maximum peak inside 1.5 s) before function approximation to suppress noise. Pupil constriction, i.e., response to brightening, was not modeled, as LC activity mainly relates to dilation.

Individual response functions for pupil dilation (IRFs; Fig. 2) were derived from the continuous illuminance task. In line with previous work^34^, we assumed that the neuromuscular transmission and biomechanics of the pupil response can be parsimoniously described as a combination of two linear time-invariant (LTI) systems. Furthermore, we assumed that illuminance changes engender an almost instantaneous neural input into the pupillary system. This allows deriving the system's IRF from illuminance responses. In the context of oddball responses, we were only interested in pupil dilation. Hence, we only derived the IRF for pupil dilation, i.e., in response to decreases in illuminance.

For each trial, we subtracted the first data point as a baseline value. Next, we brought responses to different illuminance decreases onto the same scale, by using a steady-state response model derived in our previous work^34^. This model describes the steady-state relationship between pupil size and illuminance levels with an exponential function: *d(E*_*v*_*)* = *C* + A*exp*(BE*_*v*_*)*, where d is the peak normalized steady-state pupil diameter and E_v_ is illuminance in lx; parameters were taken from our previous work: *A* = 32.56, *B* = − 0.48/lx^-1^, *C* = − 1.03. We divided each baseline-corrected segment by the difference of the predicted steady-state pupil response for the illuminance levels before and after change in illuminance. These corrected mean responses to sudden decreases in continuous illuminance were then averaged over all segments and low-pass filtered (cutoff: 4 Hz) to increase the signal-to-noise ratio for each participant. The IRF was finally obtained by fitting the time derivative of the mean response with a gamma probability density function (pdf), using ordinary least-squares minimization and a Nelder-Mead simplex search algorithm (*d*(*t*) = *c*(*θ*^k^Γ(*k*))^−1^*t*^*k*−1^exp(−*t*/*θ*), where *d* is the peak normalized pupil diameter, *t* is time, Γ is the gamma function, and *c, θ* and* k* are free parameters; time window: 5 s). To avoid an impact of noise subsequent to the initial pupil dilation of interest, the time derivative was zeroed at the trough after the maximum peak inside the first 1.5 s of the trial. Function approximation was used to further reduce noise in the empirically derived IRF and to yield a low-dimensional parametrization for intersubject comparison. We note that parameters of this IRF are not meant to directly correspond to biophysical properties. Parameter estimation was not found to be dependent on starting values for the search algorithm.

### Modeling LC activity

Building on our previous approach^34^, we estimated LC activity underlying pupillary dilation in the auditory oddball paradigm. To derive the continuous pupil dilation response to oddball sounds, we averaged, for each participant, the pupil response after oddball sounds and subtracted the averaged response after standard sounds. Our previous work had revealed that with an intertrial interval of 2 s (Fig. 9E in Korn and Bach^34^), the average response to oddballs can last longer than 2 s, requiring a longer time window. However, the response to the next stimulus begins after 2 s, and on a participant-by-participant level, this response is not always averaged out and can bias the estimation of the response to the initial sound. This motivated the following approach. We used the oddball response over 4 s after sound onset, detected the peak value inside the trial (2 s), and zeroed the data after the trough following this peak.

These data were then fitted with a pupillary response model that convolves a neural input with each participant’s estimated IRF. As in our previous work^34^, we described the neural input with a gamma pdf with four free parameters (x-offset, shape, scale and amplitude). Importantly, using a gamma model allows for the extraction of shape-related information which may otherwise be lost; an approach which has been shown feasible in the analysis of event-related neural potentials^45^. We fitted the model using ordinary least-squares minimization and a Nelder-Mead simplex search algorithm. Parameters were constrained such that the temporal mean of the gamma neural input was smaller than the time window over which the model was evaluated (4 s). Different from the IRF model, initial parameter values did affect parameter estimation. Thus, we used a random search with n = 1000 sets of initial values per participant, and retained the best fit. Also different from IRF, we specified an x-offset as a fourth free parameter with an upper limit of 500 ms to allow for different latencies of the neural input. One healthy participant’s pupillary response could not be fitted satisfactorily by this approach (visibly large difference between convolved input and observed response as well as poor fit of IRF) and was excluded from further analysis. For a second participant, who did not partake in the illuminance task, we used the group-level response function parameters estimated in our previous study^34^ with the same illuminance task in place of the IRF.

### Microsaccade detection and rate calculation

Gaze data recorded at 500 Hz for both eyes by the EyeLink 1000 system was first filtered using a LOWESS (Locally Weighted Scatterplot Smoothing) regression in Matlab with a span of 25 ms, optimal for microsaccade detection^46^. Detection of microsaccades was next performed using the Microsaccade Toolbox in R, which uses a velocity threshold algorithm to identify (micro)saccades in the eye-tracking data^47^. Microsaccades were further defined to have an amplitude between 0.08° and 1.5° and peak velocity between 8 and 150°/s, while microsaccade rate in Hz was calculated using a Gaussian window with a step size of 2 ms and sigma set to 50 ms^48^. Baseline-correction was performed trial-wise using the mean rate during the last 200 ms before the stimulus, i.e., before oddball or standard tone in the auditory paradigm and before darkening stimulus in the illuminance task.

To quantify microsaccadic inhibition in response to oddballs over standard tones, we measured peak inhibition as well as inhibition averaged across a time window encompassing full inhibition. Peak inhibition was defined as the time bin starting 50 ms before until 50 ms after the minimum signal in the first 600 ms. The time window for the full response was defined as starting from stimulus onset and lasting until signal rebound for the mean signal, which was located at 660 ms.

### Statistical analyses

Results were analyzed using descriptive quantitative measures adapted to the single-case experimental design. Specifically, we focused on establishing the probability that BG was an outlier in our sample. Since the gamma parameters themselves have no biophysical or neural interpretation, we summarized them into two biologically meaningful statistics of the gamma pdf, response mean time (= shape × scale for IRF and shape × scale + x-offset for modeled LC activity) and response excess kurtosis (= 6/shape), as measures of its timing and shape. Excess kurtosis, a metric of ‘tailedness’ of a probability distribution compared to the normal distribution, is also related to how pronounced the peak of a gamma distribution is. The distributions of mean and excess kurtosis were shown to be compatible with normality within the healthy sample using the Kolmogorov–Smirnov test. Comparisons between patient and healthy controls were made then using the Bayesian test for single case assessment developed by Crawford and Garthwaite^49^ and computed with the R library ‘psycho’^50^.

## Results

### Pupillary responses to changes in illuminance

**Figure 3:**
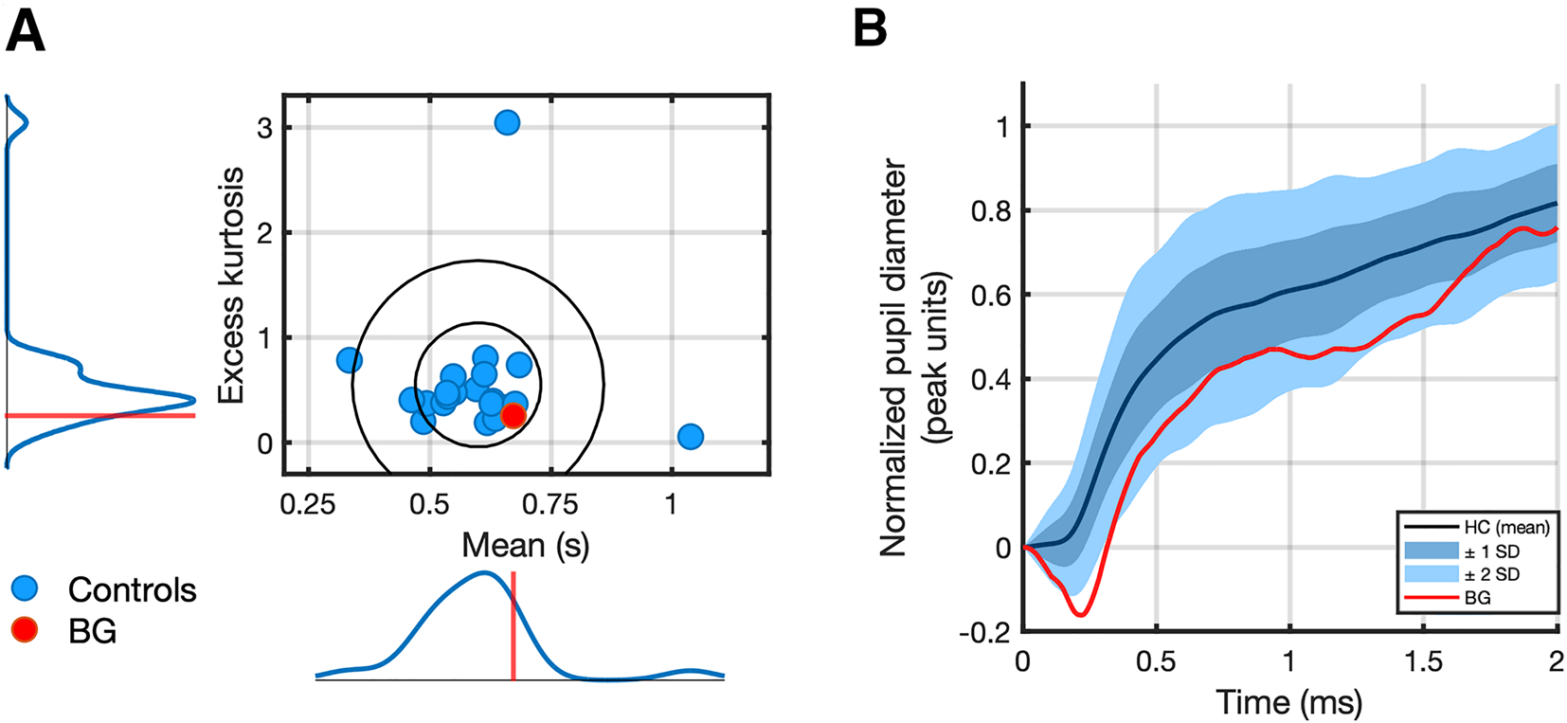
(**A**) Distributions of excess kurtosis and mean with standard deviational ellipses (± 1 SD and ± 2 SD) for individual pupillary dilation response functions for BG and healthy controls (n = 21). (**B**) Mean normalized pupil dilation response to decreases in illuminance for entire sample. ± 1 SD and ± 2 SD are shaded in darker and lighter blue, respectively. Pupil dilation timeseries for BG, overlaid in red, exhibits an initial constriction lower than -2 SD.

To rule out alterations in BG's pupillary system, we first compared illuminance responses between BG and healthy controls. Averaged responses are shown in Fig. 3. To reduce dimensionality for statistical analysis, we compared measures of timing and shape of the estimated IRF. This revealed that BG's IRF was not an outlier from the control sample. Mean ± SD of the IRF mean time for healthy controls was 596 ms ± 131 ms, and 672 ms for BG. Mean ± SD of IRF excess kurtosis for healthy controls was 0.565 ± 0.601 and (BG): 0.258. Bayesian test for single case assessment provided point estimates of BG’s IRF mean time and excess kurtosis percent rank within the controls sample, which were at 71.15% and 31.04%, respectively, and thus not statistically different from controls (p = 0.289; p = 0.310).

Inspection of BG's direct illuminance responses revealed marked pupil constriction prior to dilation in the first 500 ms of her response to illuminance reduction. This initial constriction was more pronounced than in the other participants (Fig. 3B). As it was not captured by the gamma pdf fitted to the pupil dilation response time derivative, the constriction did not affect the response functions parameters.

### Inferred LC activity in response to auditory oddball stimuli

**Figure 4:**
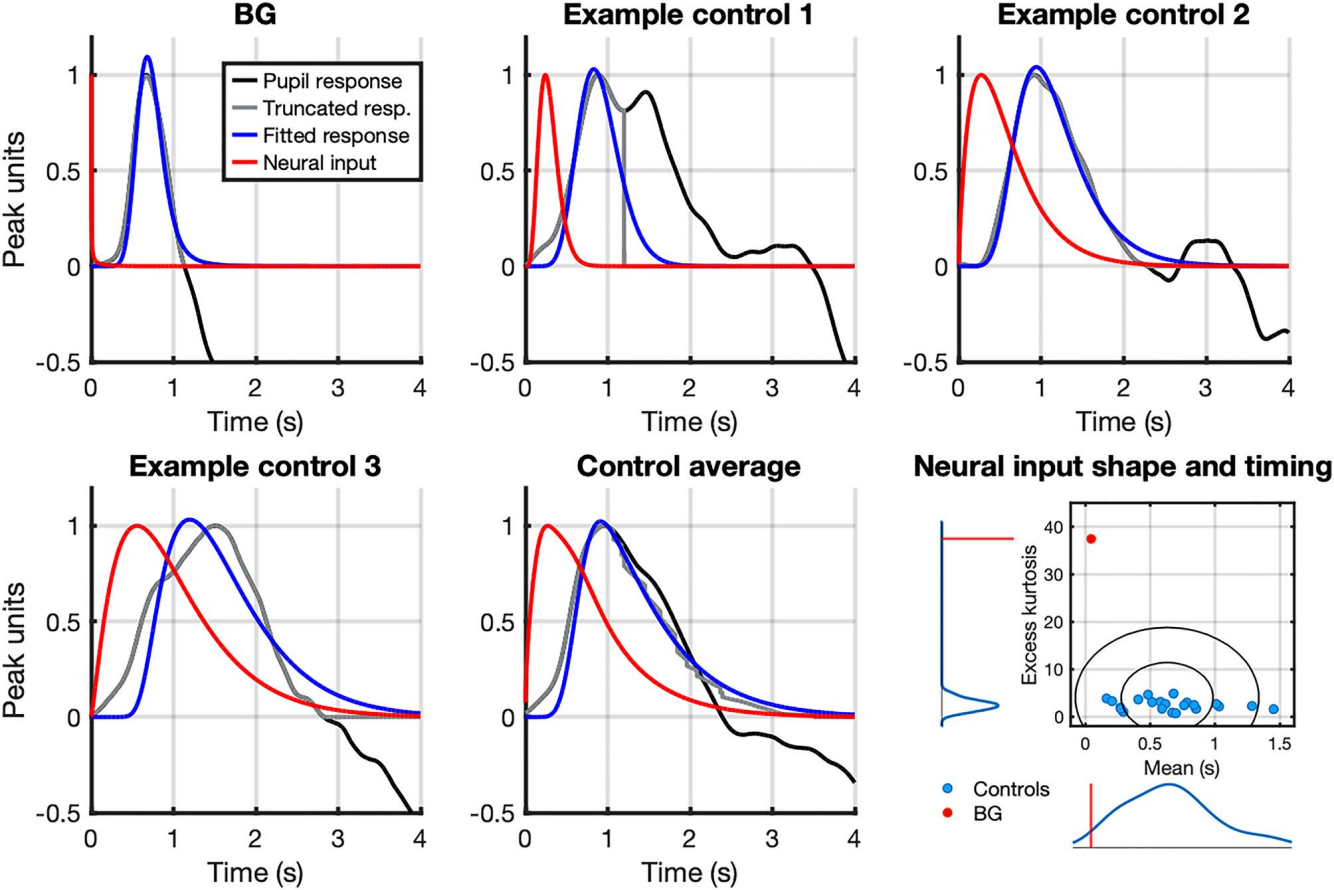
Pre-processed pupillary responses to oddball minus responses to standard tone (black) and fitted convolution (blue) of inferred neural input (red) with individual pupillary response function (not shown) for subject with Urbach-Wiethe syndrome (BG), three healthy controls and control average (n = 22). Fitting the convolution to the truncated pupillary response (after first peak, gray) avoids variability after the initial dilation. Distributions of excess kurtosis and mean process times with standard deviational ellipses for estimated neural input gamma pdfs for BG and controls are displayed on the last panel (lower right).

Next, we compared pupil responses to oddball sounds. To reduce dimensionality and to provide an interpretable summary of the individual pupil responses, we estimated LC neural output from the data and compared parameters of this neural function. The estimated neural input exhibited a premature, abbreviated, impulse-shaped form exclusively for BG and not for the healthy controls (Fig. 4), whose inputs uniformly assumed a wave pulse-like form. This visual difference between the inputs was confirmed by an analysis of their mean and excess kurtosis. BG's neural response mean time was markedly lower than for any control participant (43 ms; mean ± SD for healthy controls: 657 ms ± 337 ms) and her neural response excess kurtosis was increased (37.482; mean ± SD for healthy controls: 2.513 ± 1.122). Bayesian test for single case assessment gave a point estimate of the mean lower than 95.55% of the control population and excess kurtosis higher than 100.00% of the control population. Both mean time (p = 0.045) and excess kurtosis were (p < 0.001) were significantly different from controls after Holm-Bonferroni correction.

**Figure 5:**
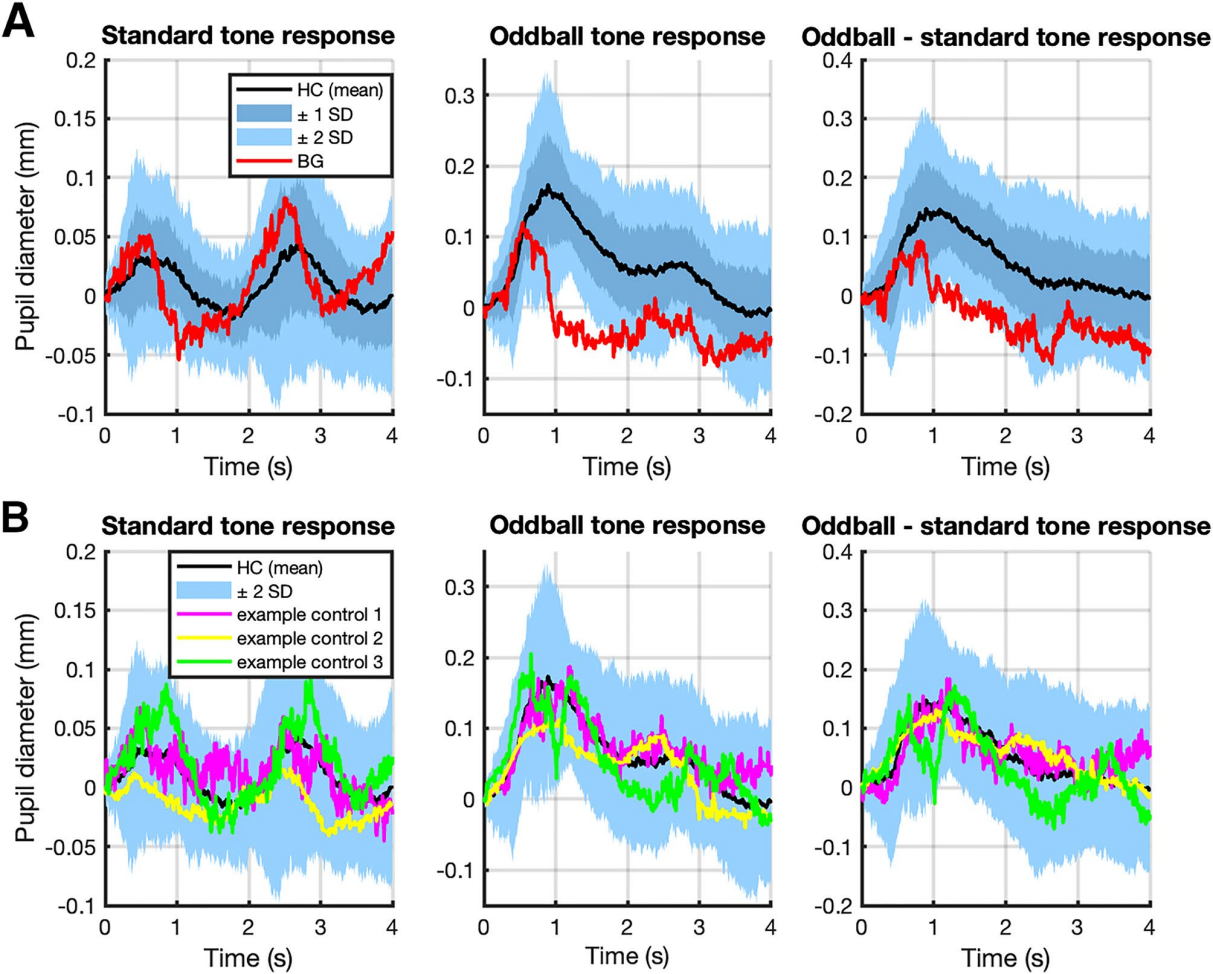
(**A**) Raw pupil response data for BG vs. mean control response (± 1 and 2 SD) in auditory oddball task. Panels depict response to standard tone, oddball tone, and oddball—standard tone from left to right. (**B**) Pupil responses for three example controls vs. mean response (± 2 SD).

An abbreviated response to oddballs could also be seen in the raw pupil responses for BG compared to controls (Fig. 5), while her baseline pupil diameter was close to the control mean (BG: 2.89 mm; mean ± SD for healthy controls: 2.82 mm ± 0.45 mm).

To exclude the possibility that the predilatory pupil constriction that was not captured by our model (see Fig. 3B) would explain this pattern, we modeled the neural input one more time, using the time derivative of the illuminance response (zeroed after the first trough) directly instead of the fitted gamma pdf for the response function (Supplementary Figure 1). This approach did not alter the impulse-like shape nor the speed of the estimated neural input but rather accentuated it with an excess kurtosis of 70.175 and a mean of 27 ms.

In an exploratory analysis, we also investigated the time offset for LC activity. This proved to be minimal and similar for all participants (BG: 0.41 ms; mean ± SD for healthy controls: 0.46 ms ± 0.10 ms).

### Microsaccade rate and inhibition in response to darkening and auditory stimuli

**Figure 6:**
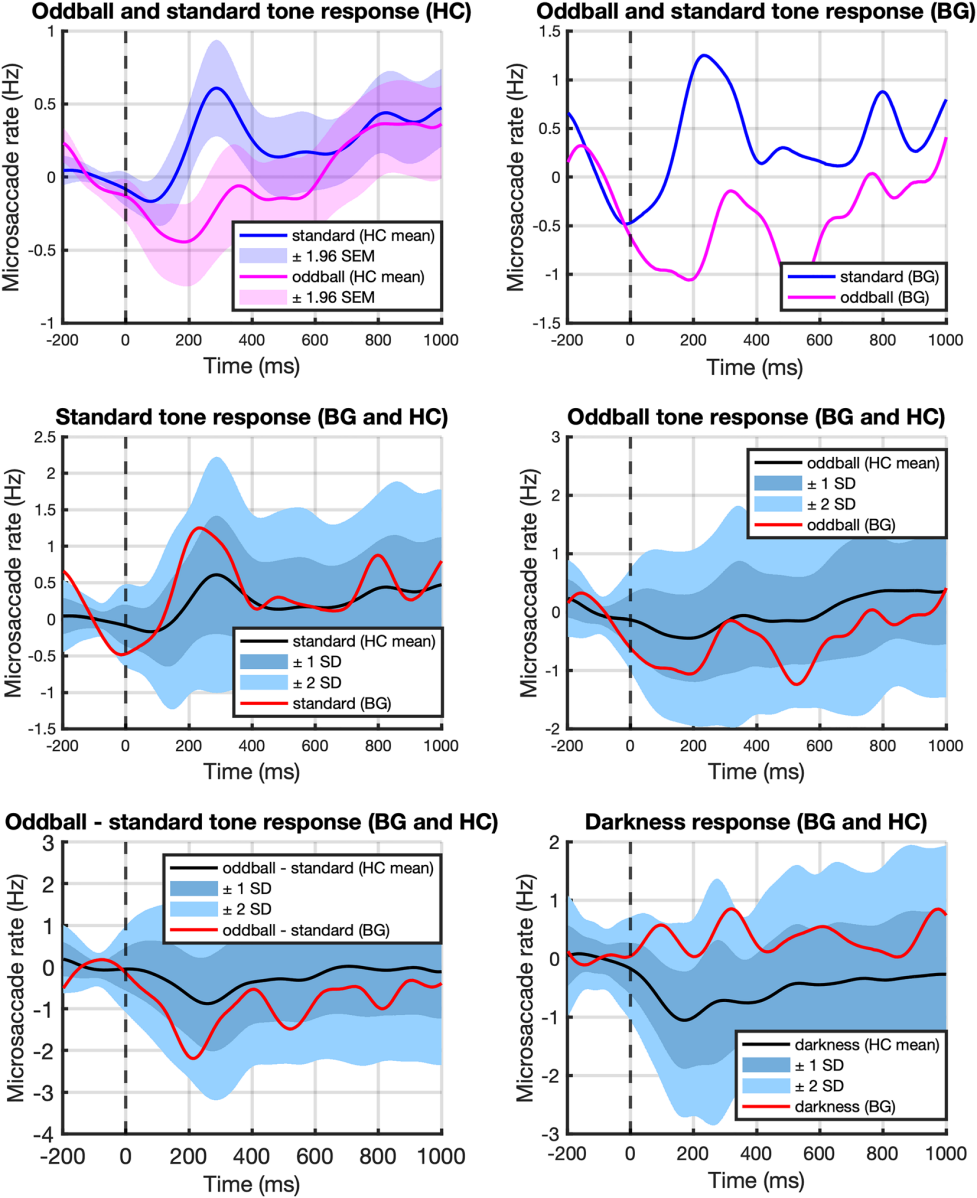
Microsaccade analysis: Microsaccade rate in response to oddball and standard tones. Increased microsaccadic inhibition in response to auditory oddballs can be observed in both healthy controls and BG. Her responses do not differ significantly from the control sample in any condition. While less pronounced, the microsaccadic response to darkness in the illuminance paradigm lies within the sample norm for BG.

To narrow down potential sources of the observed difference in pupil response to oddballs, a secondary microsaccade analysis was performed (Fig. 6). This analysis revealed no significant deviation from the norm for BG in terms of microsaccadic inhibition within the oddball paradigm. Specifically, controls as well as BG exhibited stronger inhibition in response to oddballs than standard tones. Accordingly, neither peak inhibition (BG: −2.00 Hz, mean ± SD for controls: −1.12 Hz ± 0.53 Hz) nor inhibition averaged over time of the oddball—standard response from stimulus onset until mean rebound time (BG: −1.09 Hz, mean ± SD for controls: −0.38 Hz ± 0.65 Hz) were different for BG.

The response to the darkness stimulus in the illuminance task as seen in Fig. 6 was also within bounds of the sample norm.

## Discussion

Numerous studies have examined the separate roles of LC and amygdala in auditory surprise and novelty processing, yet research on their interaction, particularly in humans, remains sparse. Here, we used pupil dilation, which is directly influenced by LC output, to measure responses to auditory oddball targets in both healthy controls and a female subject (BG) with non-functioning amygdalae due to Urbach-Wiethe (UW) syndrome. To reduce dimensionality and provide interpretable indices, we estimated LC neural output from the data and analyzed its temporal and shape characteristics.

Two key findings emerged: First, estimated phasic LC response to oddballs was compacted to an impulse-shaped form in the patient with bilateral amygdala lesions, while extending over time in all other participants. Second, BG exhibited an increased predilatory pupillary constriction in response to rapid decrease in continuous illuminance in comparison with healthy controls. Importantly, the different oddball responses could not be satisfactorily explained by the latter discrepancy in pupil reactivity.

We were able to show that the impulse-shaped form of the estimated neural response to auditory oddballs was a clear and secluded outlier in our sample by quantifying its kurtosis, which proved significantly different from controls in a Bayesian test. This also held true for the mean of the estimated neural input, which was located markedly earlier in time for BG than for the healthy participants. Differences in onset time were not seen for the estimated neural responses.

Phasic activity in LC is frequently comprised of short bursts of a few action potentials succeeded by a continuous period of suppressed neural activity, reported at 200–500 ms^51^. This suppression is, at least in part, mediated by auto-inhibition of LC via activation of α2-adrenergic autoreceptors through local NA release^52^. One potential explanation for the abbreviated neural response seen in the present study is that reciprocal connections between LC and amygdala are required to sustain LC activity, for instance, by rescuing neural activity from rapid autoinhibition after its initial ignition by the oddball detection. Central amygdala, in particular, could be involved as it has strong inhibitory as well as corticotropin releasing factor (CRF) delivering excitatory projections to LC, which are triggered by environmental stress in rodents^53,54^. Strikingly, and in a reversal of the pattern seen here, CRF release in LC has been reported to shift the mode of LC activity from phasic to a high tonic state^55^. Conversely, the loss of inhibitory projections from amygdala to LC may account for an earlier mean of the response.

We note that minor sparing from calcification has been reported for medial and lateral parts of central amygdala, five years prior to the experiment. While calcification of amygdala is thought to be slowly progressive in UW^56^, the possibility of some viable tissue in these areas cannot be fully excluded. Interestingly, patients with basolateral amygdala (BLA) lesions but wholly intact central amygdala in a South African UW cohort (n = 5) exhibited enhanced recognition of fearful faces, likely due to disrupted BLA inhibition of central amygdala^57^. This finding, nevertheless, contrasts reports in BG, where fearful face recognition is severely impaired^36^.

In a previous study, we observed an analogous impulse-shaped estimated neural input in response to a visual target detection task (infrequent red crosses vs. distractor stream of white digits) in a healthy sample at the group-level analysis^34^, whereas the group-level response to auditory oddballs then was similar to our individual-level healthy participant responses here. Based on the shorter latency of the estimated neural input, we had previously hypothesized that the neural response to visual targets originated predominantly locally, while responses to auditory oddballs may be modulated by cortical influences. Our current study implicates the amygdala-LC circuit in such a process.

On a behavioral level, BG was still able to detect oddballs at a similarly high rate to the other participants. While this strongly suggests that amygdala (and putatively sustained LC activity) is not a sine qua non for oddball appraisal, targets in our task were easily distinguished from standard tones by design, which may not hold true for less distinctive situational features.

Phasic activity of LC was estimated by fitting the convolution of a gamma function and a pupillary response function to the pupil response data, in an approach developed in our previous study^34^. Here, we advanced this method by modelling the pupillary dilation function (i.e., in response to rapid darkening of continuous illuminance), on an individual- as opposed to group-level, using prior low-pass filtering to increase the signal-to-noise ratio.

To exclude task-independent differences in pupil reactivity in BG, we compared her individual pupil dilation response function to that of healthy controls and found it to be in relative proximity to the population mean. We did, however, observe a predilatory pupillary constriction, which was also present in a subset of healthy controls, but was more pronounced for BG and more than two standard deviations removed from the mean. As this early constriction was not modeled by the pupil dilation response function, altering or extending influence on the inferred neural input is unlikely. A follow-up analysis using the unfitted temporal derivative of the pupil response to illuminance (which included the constriction) directly as the response function did not change the impulse-like form of the estimated input (Supplementary Figure 1). Also, considering timing, the pupil constriction seen in response to darkening had reversed completely before the begin time of the pupil dilation seen in response to the auditory oddball. Un- and preprocessed pupil response to oddballs showed no initial constriction. Despite these findings, we note that we cannot completely rule out a relationship between the atypical response in the illuminance task and the oddball task in our current data.

Trivially, this early constriction in BG and other participants can be explained by accommodation and fixation dynamics initiated by appearance or disappearance of the contrasting disc during the task^58^. As pupil constriction is believed to be mainly mediated by a parasympathetic pathway, while pupil dilation is driven by sympathetic fibers (both ending in two antagonistic neuromuscular systems)^59,60^, the amplified undershoot in BG may point to a relative reduction in sympathetic tone, plausibly related to the amygdala damage, as amygdala is known to activate the autonomous nervous system via brainstem and hypothalamus^61,62^. It is worth noting that inhibition of the parasympathetic pathway has also been shown to contribute to pupil dilation, in particular, during task processing^63^. Intriguingly, pupil constriction in the context of the pupillary light response has been related to covert attentional orienting responses to relatively brighter peripheral regions^64^.

In a secondary analysis, we measured changes in microsaccade rate during both tasks. Notably, BG showed increased microsaccadic inhibition in response to auditory oddballs, in line with the pattern seen here in healthy controls and in previous studies^48,65^. Given that microsaccades are thought to be generated in the superior colliculus^66^, the combination of an atypical pupil response with intact microsaccadic reactivity strengthens the case for a specific dysfunction in LC^67^.

An evident limitation of the current paper is the reliance on a single participant with UW syndrome. Nevertheless, the fact that BG has been studied extensively in previous studies from our and other groups and that she exhibits near total bilateral amygdala destruction with no involvement of adjacent brain structures is a substantial advantage. Furthermore, the correspondence of the inferred LC response to auditory oddballs in BG to a group-level response in healthy individuals to visual oddballs in a previous study using analogous methods, serves as circumstantial evidence for the credibility of the single-subject behavior of the estimated response. We note, however, that while pupil measurements are correlated with LC activity in humans^68^, we cannot exclude causal influence of other brain areas (in combination with amygdala) on the altered pupil reactivity. Inferior and superior colliculi as well as cingulate cortex can influence LC through direct connections and do also relate to pupil dilation, albeit with some time delay as shown in nonhuman primates^11^. A superior colliculi-centered network, in particular, has been implicated in pupil responses in relation to orienting behavior, while responses in relation to arousal are thought to pertain to the LC-centered circuit^69^. In a study in mice, moreover, rapid pupil dilations were shown to be tightly linked to phasic NA release, while longer-lasting pupil dilations were associated with acetylcholine activity^10^; a neurotransmitter which is produced in the basal forebrain, which in turn receives input from amygdala in both rodents^70^ and non-human primates^71^. Recently, it has been further argued that coupling of LC and pupil size may vary across brain states in mice^72^. However, it is important to note that pupil response to evoked LC activation exhibit considerable differences in latency in rodents when compared to nonhuman primates^11,73^, warranting caution when directly applying findings from rodents to humans.

Lastly, we note that motor responses or go-trials in general have been shown to contribute to pupil dilation, which has rarely been accounted for in oddball tasks^74,75^. Under the assumption of a causal effect on pupil diameter independent of LC activity, controlling for such a variable may, in theory, increase precision of the estimated relationship between the other two variables^76^. Interestingly, a recent study has highlighted dynamical differences in pupil response between active and passive detection of temporal regularity of sounds, which were unrelated to motor response^77^. Specifically, pupil dilation responses were seen at the beginning and end of random stimulus patterns when these patterns were also targets but only at beginning when not. We note that opting for an active paradigm here precludes disentangling these processes.

In summary, phasic LC response to auditory oddball targets estimated from pupil dilation recordings was revealed to be shortened in a female subject with non-functioning amygdalae compared to healthy controls, while behavioral detection of these targets remained intact. Our paper suggests that communication with amygdala modulates and sustains LC activity over time and during the detection of anomalous acoustic events. It also provides further evidence for the involvement of amygdala in deviant sound detection and processing, while indicating that it is not essentially required for it.

### Supplementary Information

Supplementary Figure 1.

## Data Availability

Anonymized data for healthy controls is available on Zenodo (10.5281/zenodo.8239465)^78^. Data for the patient participant can be provided to academic entities upon request to D.R.B. under a data protection agreement.

## References

[1] Bach DR (2008). Rising sound intensity: An intrinsic warning cue activating the amygdala. Cereb. Cortex.

[2] Linden DEJ (1999). The functional neuroanatomy of target detection: An fMRI study of visual and auditory oddball tasks. Cereb. Cortex.

[3] Slabu L, Grimm S, Escera C (2012). Novelty detection in the human auditory brainstem. J. Neurosci..

[4] Carbajal GV, Malmierca MS (2018). The neuronal basis of predictive coding along the auditory pathway: From the subcortical roots to cortical deviance detection. Trends Hear..

[5] Vazey EM, Moorman DE, Aston-Jones G (2018). Phasic locus coeruleus activity regulates cortical encoding of salience information. Proc. Natl. Acad. Sci..

[6] Poe GR (2020). Locus coeruleus: A new look at the blue spot. Nat. Rev. Neurosci..

[7] Blackford JU, Buckholtz JW, Avery SN, Zald DH (2010). A unique role for the human amygdala in novelty detection. Neuroimage.

[8] Usher M, Cohen JD, Servan-Schreiber D, Rajkowski J, Aston-Jones G (1999). The role of locus coeruleus in the regulation of cognitive performance. Science.

[9] Eldar E, Cohen JD, Niv Y (2013). The effects of neural gain on attention and learning. Nat. Neurosci..

[10] Reimer J (2016). Pupil fluctuations track rapid changes in adrenergic and cholinergic activity in cortex. Nat. Commun..

[11] Joshi S, Li Y, Kalwani RM, Gold JI (2016). Relationships between pupil diameter and neuronal activity in the locus coeruleus, colliculi, and cingulate cortex. Neuron.

[12] Zerbi V (2019). Rapid reconfiguration of the functional connectome after chemogenetic locus coeruleus activation. Neuron.

[13] Waterhouse BD, Navarra RL (2019). The locus coeruleus-norepinephrine system and sensory signal processing: A historical review and current perspectives. Brain Res..

[14] McBurney-Lin J, Lu J, Zuo Y, Yang H (2019). Locus coeruleus-norepinephrine modulation of sensory processing and perception: A focused review. Neurosci. Biobehav. Rev..

[15] Martins ARO, Froemke RC (2015). Coordinated forms of noradrenergic plasticity in the locus coeruleus and primary auditory cortex. Nat. Neurosci..

[16] Phelps EA, LeDoux JE (2005). Contributions of the amygdala to emotion processing: From animal models to human behavior. Neuron.

[17] Zald DH (2003). The human amygdala and the emotional evaluation of sensory stimuli. Brain Res. Rev..

[18] Bach DR (2008). The effect of appraisal level on processing of emotional prosody in meaningless speech. Neuroimage.

[19] Anderson AK, Phelps EA (2001). Lesions of the human amygdala impair enhanced perception of emotionally salient events. Nature.

[20] Bach DR, Talmi D, Hurlemann R, Patin A, Dolan RJ (2011). Automatic relevance detection in the absence of a functional amygdala. Neuropsychologia.

[21] Bach DR, Hurlemann R, Dolan RJ (2015). Impaired threat prioritisation after selective bilateral amygdala lesions. Cortex.

[22] Roesch MR, Esber GR, Li J, Daw ND, Schoenbaum G (2012). Surprise! Neural correlates of Pearce-Hall and Rescorla-Wagner coexist within the brain. Eur. J. Neurosci..

[23] McCall JG (2017). Locus coeruleus to basolateral amygdala noradrenergic projections promote anxiety-like behavior. Elife.

[24] McCall JG (2015). CRH engagement of the locus coeruleus noradrenergic system mediates stress-induced anxiety. Neuron.

[25] Naegeli C (2018). Locus coeruleus activity mediates hyperresponsiveness in posttraumatic stress disorder. Biol. Psychiat..

[26] Morris LS (2020). Sub-millimeter variation in human locus coeruleus is associated with dimensional measures of psychopathology: An in vivo ultra-high field 7-Tesla MRI study. NeuroImage Clin..

[27] Klimek V (1997). Reduced levels of norepinephrine transporters in the locus coeruleus in major depression. J. Neurosci..

[28] Etkin A, Prater KE, Hoeft F, Menon V, Schatzberg AF (2010). Failure of anterior cingulate activation and connectivity with the amygdala during implicit regulation of emotional processing in generalized anxiety disorder. Am. J. Psychiatry.

[29] Grueschow M (2021). Real-world stress resilience is associated with the responsivity of the locus coeruleus. Nat. Commun..

[30] Fast CD, McGann JP (2017). Amygdalar gating of early sensory processing through interactions with locus coeruleus. J. Neurosci..

[31] Näätänen R, Gaillard AWK, Mäntysalo S (1978). Early selective-attention effect on evoked potential reinterpreted. Acta Physiol. (Oxf.).

[32] Swick D, Pineda JA, Schacher S, Foote SL (1994). Locus coeruleus neuronal activity in awake monkeys: Relationship to auditory P300-like potentials and spontaneous EEG. Exp. Brain Res..

[33] Camalier CR, Scarim K, Mishkin M, Averbeck BB (2019). A Comparison of auditory oddball responses in dorsolateral prefrontal cortex, basolateral amygdala, and auditory cortex of macaque. J. Cogn. Neurosci..

[34] Korn CW, Bach DR (2016). A solid frame for the window on cognition: Modeling event-related pupil responses. J. Vis..

[35] Korn CW, Staib M, Tzovara A, Castegnetti G, Bach DR (2017). A pupil size response model to assess fear learning. Psychophysiology.

[36] Becker B (2012). Fear processing and social networking in the absence of a functional amygdala. Biol. Psychiat..

[37] Hurlemann R (2010). Oxytocin enhances amygdala-dependent, socially reinforced learning and emotional empathy in humans. J. Neurosci..

[38] Talmi D, Hurlemann R, Patin A, Dolan RJ (2010). Framing effect following bilateral amygdala lesion. Neuropsychologia.

[39] Hurlemann R (2007). Amygdala control of emotion-induced forgetting and remembering: Evidence from Urbach-Wiethe disease. Neuropsychologia.

[40] Doppelhofer LM, Hurlemann R, Bach DR, Korn CW (2021). Social motives in a patient with bilateral selective amygdala lesions: Shift in prosocial motivation but not in social value orientation. Neuropsychologia.

[41] Bach DR, Hurlemann R, Dolan RJ (2013). Unimpaired discrimination of fearful prosody after amygdala lesion. Neuropsychologia.

[42] Scheele D (2019). Treatment-resistant depression and ketamine response in a patient with bilateral amygdala damage. Am. J. Psychiatry.

[43] Kret ME, Sjak-Shie EE (2019). Preprocessing pupil size data: Guidelines and code. Behav. Res. Methods.

[44] Hayes TR, Petrov AA (2016). Mapping and correcting the influence of gaze position on pupil size measurements. Behav. Res. Methods.

[45] Kummer K, Dummel S, Bode S, Stahl J (2020). The gamma model analysis (GMA): Introducing a novel scoring method for the shape of components of the event-related potential. J. Neurosci. Methods.

[46] Yablonski M, Polat U, Bonneh YS, Ben-Shachar M (2017). Microsaccades are sensitive to word structure: A novel approach to study language processing. Sci. Rep..

[47] Engbert R, Mergenthaler K, Sinn P, Pikovsky A (2011). An integrated model of fixational eye movements and microsaccades. Proc. Natl. Acad. Sci. U. S. A..

[48] Kadosh O, Bonneh YS (2022). Involuntary oculomotor inhibition markers of saliency and deviance in response to auditory sequences. J. Vis..

[49] Crawford JR, Garthwaite PH (2007). Comparison of a single case to a control or normative sample in neuropsychology: Development of a Bayesian approach. Cogn. Neuropsychol..

[50] Makowski D (2018). The psycho Package: An efficient and publishing-oriented workflow for psychological science. J. Open Sourc. Softw..

[51] Devilbiss DM, Waterhouse BD (2011). Phasic and tonic patterns of locus coeruleus output differentially modulate sensory network function in the awake rat. J. Neurophysiol..

[52] Janitzky K (2020). Impaired phasic discharge of locus coeruleus neurons based on persistent high tonic discharge—a new hypothesis with potential implications for neurodegenerative diseases. Front. Neurol..

[53] Berridge CW, Waterhouse BD (2003). The locus coeruleus–noradrenergic system: Modulation of behavioral state and state-dependent cognitive processes. Brain Res. Rev..

[54] Van Bockstaele EJ, Colago EEO, Valentino RJ (1998). Amygdaloid corticotropin-releasing factor targets locus coeruleus dendrites: Substrate for the co-ordination of emotional and cognitive limbs of the stress response. J. Neuroendocrinol..

[55] Snyder K, Wang W-W, Han R, McFadden K, Valentino RJ (2012). Corticotropin-releasing factor in the norepinephrine nucleus, locus coeruleus, facilitates behavioral flexibility. Neuropsychopharmacology.

[56] Koen N (2016). Translational neuroscience of basolateral amygdala lesions: Studies of urbach-wiethe disease. J. Neurosci. Res..

[57] Terburg D (2012). Hypervigilance for fear after basolateral amygdala damage in humans. Transl. Psychiatry.

[58] Gramatikov BI, Irsch K, Guyton D (2014). Optimal timing of retinal scanning during dark adaptation, in the presence of fixation on a target: The role of pupil size dynamics. J. Biomed. Opt..

[59] Mathôt S (2018). Pupillometry: Psychology, physiology, and function. J. Cogn..

[60] McDougal, D. H. & Gamlin, P. D. R. In *The Senses: A Comprehensive Reference* (eds Richard H. Masland *et al.*) 521–536 (Academic Press, 2008).

[61] LeDoux JE (2000). Emotion circuits in the brain. Annu. Rev. Neurosci..

[62] Yang TT (2007). Increased amygdala activation is related to heart rate during emotion processing in adolescent subjects. Neurosci. Lett..

[63] Steinhauer SR, Siegle GJ, Condray R, Pless M (2004). Sympathetic and parasympathetic innervation of pupillary dilation during sustained processing. Int. J. Psychophysiol..

[64] Mathôt S, van der Linden L, Grainger J, Vitu F (2013). The pupillary light response reveals the focus of covert visual attention. PLoS ONE.

[65] Andreas W, Ralf E, Erich S (2014). Microsaccadic responses indicate fast categorization of sounds: A novel approach to study auditory cognition. J. Neurosci..

[66] Hafed ZM, Goffart L, Krauzlis RJ (2009). A neural mechanism for microsaccade generation in the primate superior colliculus. Science.

[67] Strauch C, Greiter L, Huckauf A (2018). Pupil dilation but not microsaccade rate robustly reveals decision formation. Sci. Rep..

[68] Murphy PR, O'Connell RG, O'Sullivan M, Robertson IH, Balsters JH (2014). Pupil diameter covaries with BOLD activity in human locus coeruleus. Hum. Brain Mapp..

[69] Strauch C, Wang CA, Einhauser W, Van der Stigchel S, Naber M (2022). Pupillometry as an integrated readout of distinct attentional networks. Trends Neurosci..

[70] Gielow, M. R. & Zaborszky, L. The Input-Output Relationship of the Cholinergic Basal Forebrain. *Cell Reports* **18**, 1817–1830. 10.1016/j.celrep.2017.01.060 (2017). 10.1016/j.celrep.2017.01.060 PMC5725195 28199851

[71] Russchen FT, Amaral DG, Price JL (1985). The afferent connections of the substantia innominata in the monkey, *Macaca fascicularis*. J. Compar. Neurol..

[72] Megemont M, McBurney-Lin J, Yang H (2022). Pupil diameter is not an accurate real-time readout of locus coeruleus activity. Elife.

[73] Liu Y, Rodenkirch C, Moskowitz N, Schriver B, Wang Q (2017). Dynamic lateralization of pupil dilation evoked by locus coeruleus activation results from sympathetic, not parasympathetic, contributions. Cell Rep..

[74] Richer F, Beatty J (1985). Pupillary dilations in movement preparation and execution. Psychophysiology.

[75] Strauch C, Koniakowsky I, Huckauf A (2020). Decision making and oddball effects on pupil size: Evidence for a sequential process. J. Cogn..

[76] Cinelli C, Forney A, Pearl J (2022). A crash course in good and bad controls. Sociol. Methods Res..

[77] Zhao S (2019). Pupil-linked phasic arousal evoked by violation but not emergence of regularity within rapid sound sequences. Nat. Commun..

[78] Abivardi, A. *et al.* PsPM-AOB_UW: Eye tracker (including pupillometry) measurements from an auditory oddball and a luminance task. (2023). 10.5281/zenodo.8239465.

