# Supplementary material for: Acceleration of inferred neural responses to oddball targets in an individual with bilateral amygdala lesion compared to healthy controls
Source: Sci Rep. 2023 Sep 4;13:14550. doi: 10.1038/s41598-023-41357-1 (PMC10477323; doi:10.1038/s41598-023-41357-1)
Supplement: Supplementary file 1 — Supplementary Figure 1. [file 41598_2023_41357_MOESM1_ESM.pdf]

Supplementary material

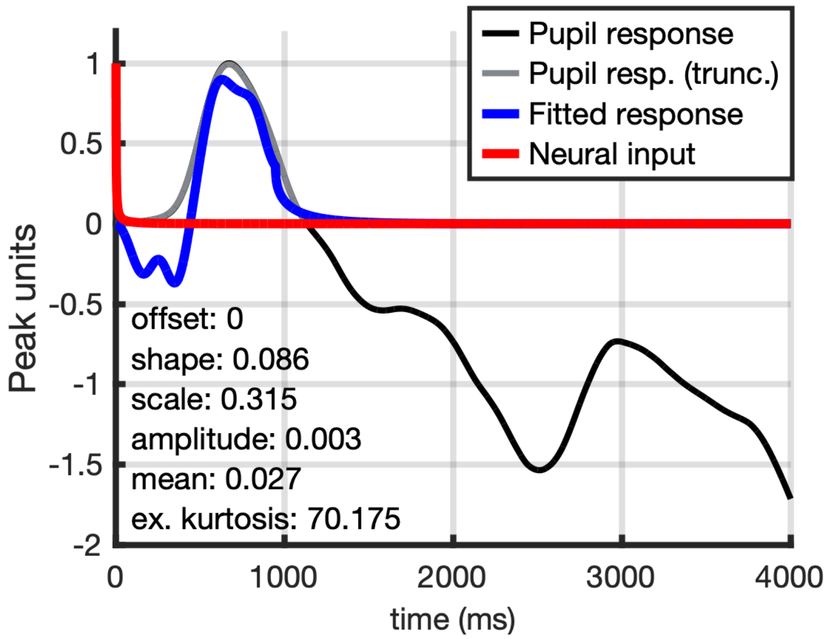

Supplementary figure 1. Estimation of neural input using raw pupil response to darkness (which includes the early constriction) instead of IRF from task.
